# Extended‐Interval Dosing and Discontinuation of Dupilumab in Atopic Dermatitis: A Real‐World Retrospective Study

**DOI:** 10.1111/1346-8138.70350

**Published:** 2026-06-11

**Authors:** Ryo Saito, Wataru Sasaki, Haruka Watanabe, Momoka Hashimoto, Rina Kamigaki, Kento Nagiri, Akio Tanaka

**Affiliations:** ^1^ Department of Dermatology, Graduate School of Biomedical and Health Sciences Hiroshima University Hiroshima Japan

**Keywords:** atopic dermatitis, dupilumab, topical steroids

## Abstract

This retrospective study evaluated the clinical courses and treatment patterns of 81 patients with atopic dermatitis (AD) treated with dupilumab at Hiroshima University Hospital. Patients were categorized into six groups based on their clinical outcomes, including those who continued treatment, achieved remission and discontinued, or discontinued due to adverse effects or insufficient efficacy. Of the 81 patients, 38 (47%) continued treatment throughout the study period, and 16 (20%) achieved remission and discontinued treatment without relapse. Analysis of baseline severity using EASI and TARC levels revealed no significant differences between these groups, suggesting that baseline severity does not predict long‐term therapeutic outcomes. Regarding concomitant topical therapy, although the potency of topical corticosteroids (TCS) remained unchanged in most patients, the total dosage was significantly reduced by a mean of 71.3%, reflecting a successful transition to proactive therapy or a reduced affected body surface area. Furthermore, this study investigated the feasibility of extending dupilumab dosing intervals. Nearly half of the patients extended their intervals including every 3 weeks (Q3W), Q4W, and up to Q8W by 6 months without exacerbation. By 12 months, 22 of the 40 evaluable patients successfully transitioned to extended intervals up to Q12W. These findings suggest that baseline disease severity does not necessarily predict the long‐term clinical course of dupilumab therapy in patients with AD. Although TCS potency is often maintained to ensure stability, the dosage can be drastically reduced. Furthermore, a subset of patients can discontinue or extend the dosing intervals, and the optimal timing for extending intervals is more flexible and can be initiated earlier than currently suggested by previous reports.

## 1 Introduction

Dupilumab is a human monoclonal antibody against interleukin (IL) 4 receptor α to inhibit IL‐4 and IL‐13 signaling. Dupilumab is an established therapy for moderate‐to‐severe atopic dermatitis (AD) [1]. However, the optimal long‐term treatment strategy remains to be determined. It is uncertain whether the dosing intervals can be extended or whether treatment can be discontinued after disease control is achieved. Although several studies have explored extended dosing regimens, evidence from real‐world clinical practices remains limited. We investigated the clinical course of patients treated with dupilumab at our institution, focusing on the feasibility of extending the dosing interval and treatment discontinuation in these patients.

## 2 Methods

### 2.1 Study Population and Data Collection

**FIGURE 1:**
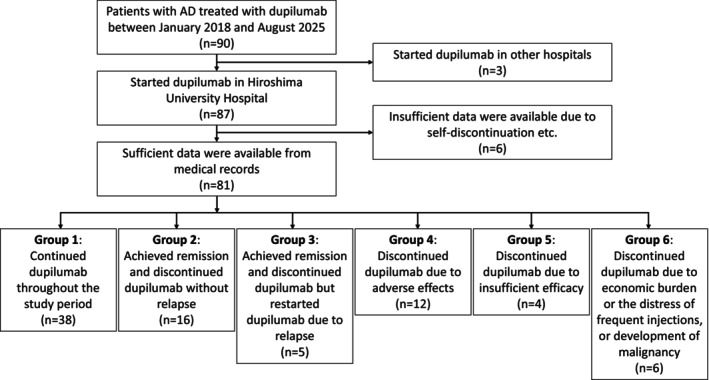
Criteria for inclusion and grouping of patients.

Data were collected retrospectively from the medical records of Hiroshima University Hospital between January 2018 and August 2025. A total of 90 patients with AD were prescribed dupilumab treatment. Of these, 87 patients who started treatment at our hospital were initially enrolled, and 6 patients were excluded from the analysis due to insufficient data, leaving 81 patients for the final analysis (Figure 1). We investigated the dosing interval of dupilumab and the proportion of patients who achieved an interval extension of more than 2, 4, 8, and 12 weeks at 1, 6, and 12 months. The extension was performed when patients achieved an IGA score of 0, 1, or 2, and a consensus was reached between the physician and the patient, taking into account the stability of AD, economic burden, and side effects which were mild enough not to warrant discontinuation of dupilumab. The amount of prescribed topical corticosteroids (TCS) was compared before and during dupilumab treatment. The daily dose was calculated by dividing the total amount of prescribed topical steroids (g) by the number of days for two distinct periods: the 6 months prior to dupilumab initiation and months 6–12 of treatment. Remission was defined as discontinuation of dupilumab after satisfactory disease control (IGA 0 or 1) without relapse requiring re‐initiation of systemic therapy during the follow‐up period.

### 2.2 Ethics Statement

The Research Ethics Committee of Hiroshima University approved the study protocol in accordance with the Declaration of Helsinki. We applied an opt‐out method for this study; information regarding the study protocol and the right to refuse participation was provided via the website of our institution.

## 3 Results

Of the 81 patients, 54 were men and 27 were women. The mean age was 43.2 years (range: 6–80 years) including five pediatric patients younger than 15. Baseline disease severity was assessed using the Investigator's Global Assessment (IGA); 49 patients had an IGA score of 3 and 21 had an IGA score of 4, while data were unavailable for 11 patients. Prior to dupilumab treatment, 25 patients received systemic therapy, including oral steroids, cyclosporine, and Janus kinase inhibitors. The patients were categorized into 6 groups according to their clinical course (Figure 1). Group 1 included patients who continued dupilumab treatment throughout the study period (*n* = 38), whereas Group 2 included patients who achieved remission and discontinued dupilumab without relapse (*n* = 16). The duration of dupilumab treatment in Group 1 ranged from 63 to 2204 days (mean 946 days). In Group 2, the administration duration ranged from 27 to 1523 days (mean 452 days), and the mean observation period after discontinuation of dupilumab was 339 days (range, 21–948 days).

The mean baseline Eczema Area and Severity Index (EASI) score was 27.4, and the median was 24, with no significant differences among the groups (Figure S1a). Similarly, no significant differences were observed in Thymus and Activation‐Regulated Chemokine (TARC) levels (Figure S1b). There was no trend in the age at the start of dupilumab therapy, whereas three of four pediatric patients achieved remission (Figure S1c,d).

**FIGURE 2:**
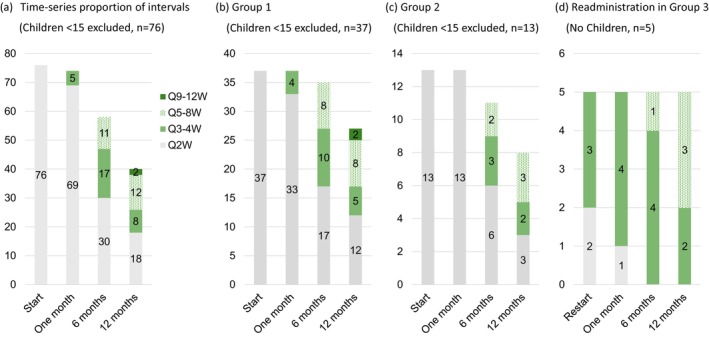
Dupilumab administration intervals. (a) All adult patients, (b) adults in group 1, (c) adults in Group 2, and (d) in Groups 3.

As dupilumab dosing regimens differ between children and adults, the interval extension analysis was restricted to adult patients older than 15 (*n* = 76). All adult patients initially received dupilumab every 2 weeks (Q2W). Nearly half of the adult patients (28 of 58) extended the dosing interval within 6 months, with some reaching Q8W dosing (Figure 2a). Among the 40 patients with at least 12 months of follow‐up, 18 remained on Q2W, whereas the others maintained extended intervals, including Q3W–Q8W, and one patient each at Q10W and Q12W (Figure 2a). The proportion of patients who achieved interval extension was similar in Groups 1 and 2 (Figure 2b,c). Three of five patients in Group 3 restarted dupilumab on Q3W, and all of them extended the intervals within 6 months of re‐initiation (Figure 2d).

**FIGURE 3:**
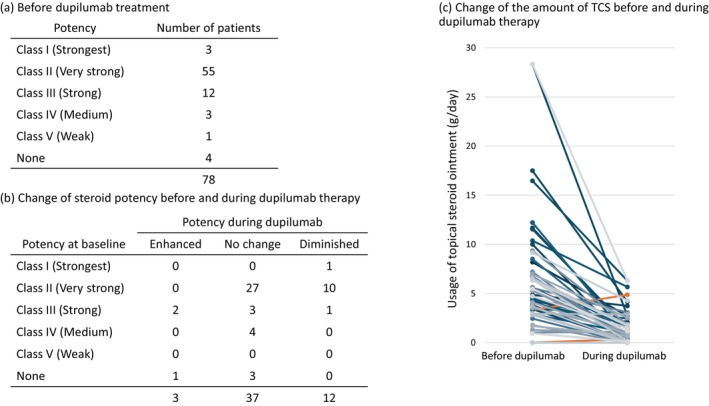
Potency and dosage of topical corticosteroids (TCS). (a) Potency of TCS before dupilumab treatment and (b) changes in potency before and during dupilumab therapy. Classification is in accordance with the guideline published by Japanese Dermatological Association [1]. (c) Comparison of TCS dosage before and during dupilumab treatment. The amount of prescribed TCS decreased in most patients (indicated in blue), increased in 2 of 52 patients (indicated in orange).

Regarding topical therapy, most patients used TCS, and class II TCS was the most commonly used in patients older than 15 (Figure 3a). We compared TCS potency and dosage before and during dupilumab therapy (at 6–12 months) in 52 patients. The potency remained unchanged in 37 patients (Figure 3b), whereas the dosage was reduced in 47 patients by a mean of 71.3% (Figure 3c).

## 4 Discussion

In this real‐world study, we found that dosing interval extension and treatment discontinuation of dupilumab were feasible in a subset of patients with AD. Our findings suggest that the baseline severity of AD was not clearly associated with the subsequent clinical course of dupilumab therapy (Figure S1a,b), consistent with recent reports indicating that EASI and NRS at baseline did not predict 75% improvement in EASI (EASI‐75) achievement at 16 weeks [2] or 6 months [3]. They also demonstrated that age and disease duration were not associated with treatment efficacy, except for a negative association between the duration of severe AD and achievement of a Patient‐Oriented Eczema Measure (POEM) score of 7 or less [2, 3]. Our data did not show a correlation between age and clinical course, although pediatric patients younger than 15 years old tended to exhibit a favorable response to dupilumab treatment (Figure S1c,d).

The management of TCS remains controversial. At our institution, we recommend continuing TCS treatment alongside dupilumab. The TCS potency remained unchanged in most patients, although the amount used decreased (Figure 3c), often due to a transition to proactive therapy or a reduction in the treated body surface area.

The standard adult regimen for dupilumab is 300 mg every 2 weeks. Extending dosage intervals after 16 weeks of Q2W treatment and achieving an IGA score of 0 or 1 or EASI‐75 resulted in a reduction in the therapeutic effect in a clinical trial [4]. In contrast, several studies have shown that extending intervals is feasible in some patients after long‐term treatment with dupilumab in a standard regimen [3, 5, 6, 7, 8, 9, 10, 11]. The injection interval up to 6 weeks resulted in sustained disease control in patients with AD with low disease activity after 52 weeks of dupilumab treatment [3]. Moreover, 12 patients who had 149 weeks of dupilumab Q2W therapy extended the dosage intervals to Q4W, and none of them showed flare‐ups after 48 weeks [7]. Based on these previous reports, the optimal timing for such extensions remains a subject of debate. Our findings suggest that the timing of interval extension can be initiated earlier than previously thought and supports individualized early stage interval extension, bridging the early failures seen in clinical trials and the very late‐stage extensions reported in previous real‐world studies. Furthermore, we demonstrated that a subset of patients sustained remission after discontinuation or long dosing intervals of dupilumab treatment. This suggests that sufficient suppression of the IL‐4/IL‐13 pathway may lead to the suppression of subclinical inflammation and immunological changes in the skin. Notably, among patients who discontinued dupilumab after achieving remission, most maintained disease control without relapses during follow‐up, suggesting that treatment discontinuation may be feasible in carefully selected patients with AD. The pathological changes occurring in these patients warrant further investigation.

This study has some limitations, as it was a retrospective, single‐center study with a small sample size. Several patients were followed up for a short term after dupilumab discontinuation (Figure S1e) and have the potential for delayed relapse. Dosing intervals were adjusted by individual dermatologists rather than using standardized criteria. Nonetheless, our findings contribute to the accumulating data on flexible dupilumab dosing, and similar reports from other institutions are warranted to further optimize the dupilumab dosing strategies.

## Conflicts of Interest

A.T. has received lecture fees from AbbVie GK, Eli Lilly Japan K.K., Tanabe Pharma Corporation, Pfizer Japan Inc., Sanofi K.K., Torii Pharmaceutical Co. Ltd., Otsuka Pharmaceutical Co. Ltd., and Maruho Co. Ltd., advisory fees from Pfizer Japan Inc., Eli Lilly Japan K.K., Sanofi K.K., and Maruho Co. Ltd., and research grants not related to the submitted work from Maruho Co. Ltd. The other authors declare no conflicts of interest.

## Supporting information


**Figure S1:** Patient characteristics. (a) EASI scores and (b) TARC levels prior to the initiation of dupilumab. (c) Age at dupilumab initiation. (d) Age at dupilumab initiation of children younger than 15. (e) Observation periods after discontinuation of dupilumab with patients in Group 2.

## Data Availability

The data that support the findings of this study are available on request from the corresponding author. The data are not publicly available due to privacy or ethical restrictions.
