# Supplementary material for: Extended‐Interval Dosing and Discontinuation of Dupilumab in Atopic Dermatitis: A Real‐World Retrospective Study
Source: J Dermatol. 2026 Jun 11;53(8):1179–83. doi: 10.1111/1346-8138.70350 (PMC13434989; doi:10.1111/1346-8138.70350)

Supplementary Figure 1. Baseline patient characteristics

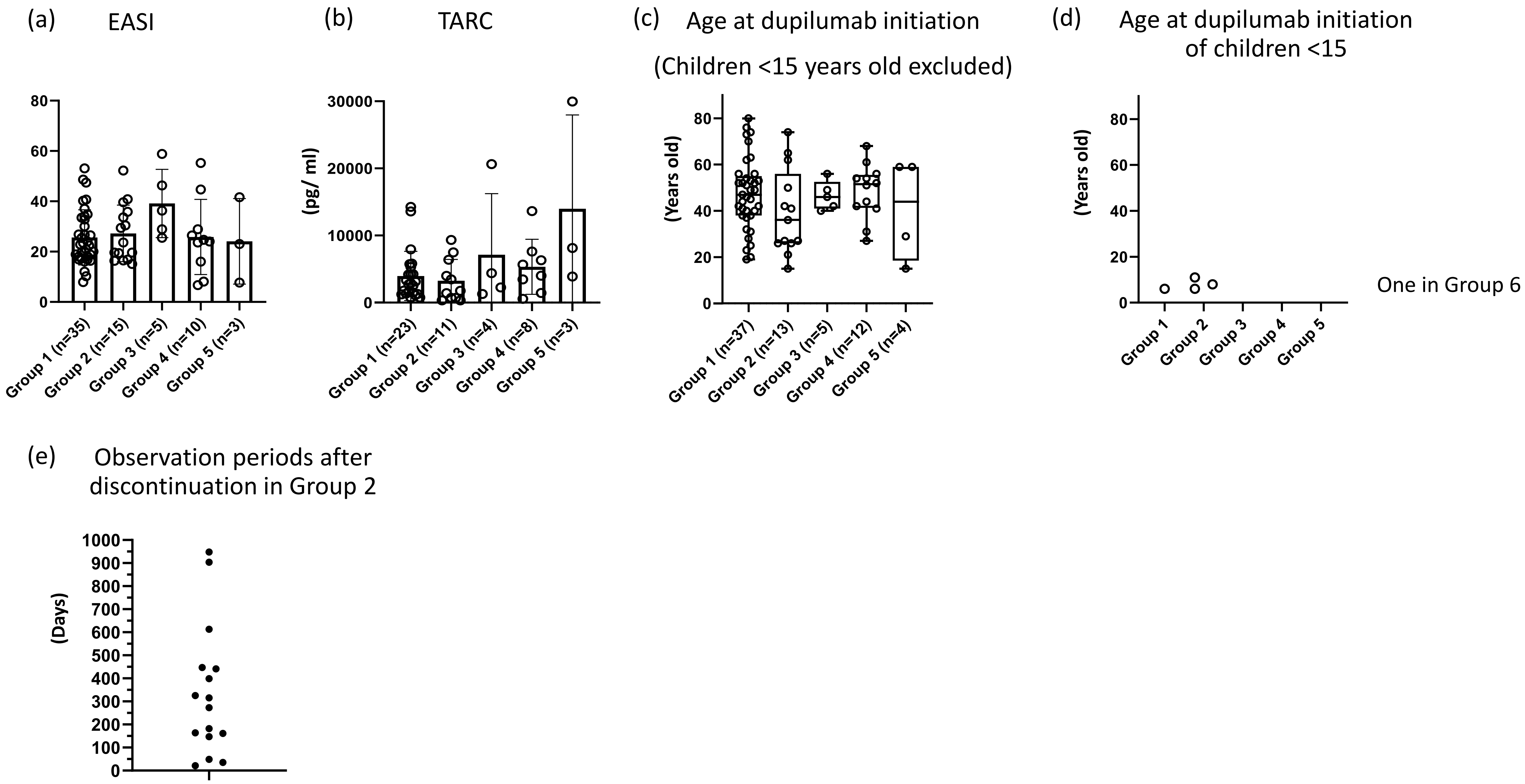

Supplement: Supplementary file 1 [file JDE-53-1179-s001.pdf]
